# Predicting clopidogrel resistance in acute ischemic stroke patients: key clinical insights and a novel diagnostic nomogram

**DOI:** 10.1186/s12883-025-04252-y

**Published:** 2025-06-03

**Authors:** Mingzhu Tang, Xiaoying Li, Gaoxian Zhong, Yaxian Dong, Tiezhu Wang, Lihua Yang, Xuanming Lai, Yongyuan Chen, Xiaolian Chen, Jinxi Zuo, Junyang Xu, Hongting Shi, Fangming Diao

**Affiliations:** 1 Department of Neurology, Institute of Neuroscience, Key Laboratory of Neurogenetics and Channelopathies of Guangdong Province and the Ministry of Education of China, The Second Affiliated Hospital, Guangzhou Medical University, No. 250, Changgang East Road, Haizhu District, Guangzhou City, 510260 China; 2 Department of Neurology, The Affiliated Shunde Hospital of Jinan University, Foshan, Guandong Province 528000 China; 3 Department of Neurology, The Fifth Affiliated Hospital of Guangzhou Medical University, Guangzhou, 510700 China

**Keywords:** Acute ischemic stroke (AIS), Clopidogrel resistance (CR), Nomogram, Protective factors, Risk factors

## Abstract

**Background:**

Clopidogrel plays an important role in the treatment of acute ischemic strokes (AIS) through antiplatelet activity. However, some patients have clopidogrel resistance (CR), which could lead to stroke recurrence and bleeding. This study aimed to explore associated factors of CR and establish a diagnostic nomogram for predicting the probability of CR in AIS patients.

**Methods:**

This retrospective study involved 692 AIS patients from the Second Affiliated Hospital of Guangzhou Medical University, treated with clopidogrel (75 mg/day for 5 ± 2 days) after admission. Platelet reactivity was evaluated using thromboelastography to measure the ADP-induced platelet inhibition ratio (ADP-PIR). Patients were classified into CR (ADP-PIR < 30%) and non-clopidogrel resistance (NCR) groups. Group comparison, followed by least absolute shrinkage and selection operator (LASSO) regression and multivariable logistic regression, was used to identify key predictors of CR. A diagnostic nomogram was developed and its performance was validated using bootstrap resampling.

**Results:**

16.76% of 692 patients experienced CR after AIS. Beta blocker use (OR: 0.47, 95% CI: 0.22–1.03, *P* = 0.058) and apolipoprotein A1 (OR: 0.17, 95% CI: 0.07–0.46, *P* < 0.001) were identified as protective factors, while unstable carotid plaque (OR: 10.65, 95% CI: 4.18–27.13, *P* < 0.001), high apolipoprotein B levels (OR: 2.35, 95% CI: 1.23–4.51, *P* = 0.01), and proton pump inhibitors use (OR: 2.09, 95% CI: 1.32–3.31, *P* = 0.002) were risk factors. Our nomogram effectively validated these factors, showing strong discrimination and clinical utility in diagnosing CR probability.

**Conclusions:**

We identified several significant CR predictors and further developed a diagnostic nomogram of CR to help clinicians choose antiplatelet drugs.

**Trial retrospectively registration:**

Trial Retrospectively registration = ChiCTR2300073944.Data: 2023-7-25. The present study was approved by the Ethics Committee of the Second Affiliated Hospital of Guangzhou Medical University.

## Introduction

Stroke remains a leading cause of mortality and disability worldwide, and ischemic strokes (IS) account for approximately 85% of all strokes [1, 2]. Recent studies have identified diverging trends in the burden of stroke: whereas the age-adjusted stroke rates have been declining in high-income countries, low- and middle-income countries continue to show a rise in stroke incidence and mortality [1, 3]. It is widely acknowledged that stroke places a significant strain on the healthcare system, not only due to the high financial costs of medical intervention and rehabilitation but also because of the emotional trauma that the family members of patients must endure [4, 5].

In effect, effective secondary prevention can reduce IS recurrence rates by as much as 80% [6]. Among the therapeutic options available, clopidogrel is one of the most widely prescribed antiplatelet agents, which irreversibly inhibits the adenosine diphosphate (ADP) receptor P2Y12 on platelets [7]. However, clopidogrel is characterized by highly variable effectiveness among individuals; about 30% of patients show clopidogrel resistance (CR), a condition associated with high on-treatment platelet reactivity (HTPR) and an increased risk for recurrent ischemic events [8, 9].

Clopidogrel is metabolized by the cytochrome P450 2C19 enzyme, and genetic polymorphisms like CYP2C19*2 and *3 alleles are strongly associated with reduced enzymatic activity and CR [10]. However, routine genetic testing is not generally recommended by the American College of Cardiology/ American Heart Association (ACC/AHA) due to insufficient evidence in prospective randomized studies. Instead, selective testing among high-risk subjects is recommended [11, 12]. Current platelet reactivity assays, such as VerifyNow and Thromboelastography, also lack standardization and reproducibility, limiting their clinical applicability [13]. The etiology of CR is multifactorial, involving not only genetic polymorphisms but also age, comorbidities such as diabetes, drug interactions, immature platelets, atherosclerosis, and inflammation [14]. The complex interplay of these factors underscores the need for integrative diagnostic approaches.

In this study, we aimed to develop a diagnostic nomogram for identifying patients at high risk of CR. By employing effective statistical techniques, such as least absolute shrinkage and selection operator (LASSO) regression and multivariable logistic regression, we integrated multiple predictors into a user-friendly tool. This nomogram could enhance clinical decision-making, optimize antiplatelet therapy, reduce stroke recurrence, and ultimately improve patient outcomes.

## Materials and methods

### Study design

This retrospective study was approved by the Ethics Committee of the Second Affiliated Hospital of Guangzhou Medical University (Approval Number: 2023-LCYJ-XS-23) and registered in the Chinese Clinical Trial Registry (Registration Number: ChiCTR2300073944). Medical records from January 2019 to December 2022 were reviewed to identify patients diagnosed with AIS at the Second Affiliated Hospital of Guangzhou Medical University [15].

**Fig. 1:**
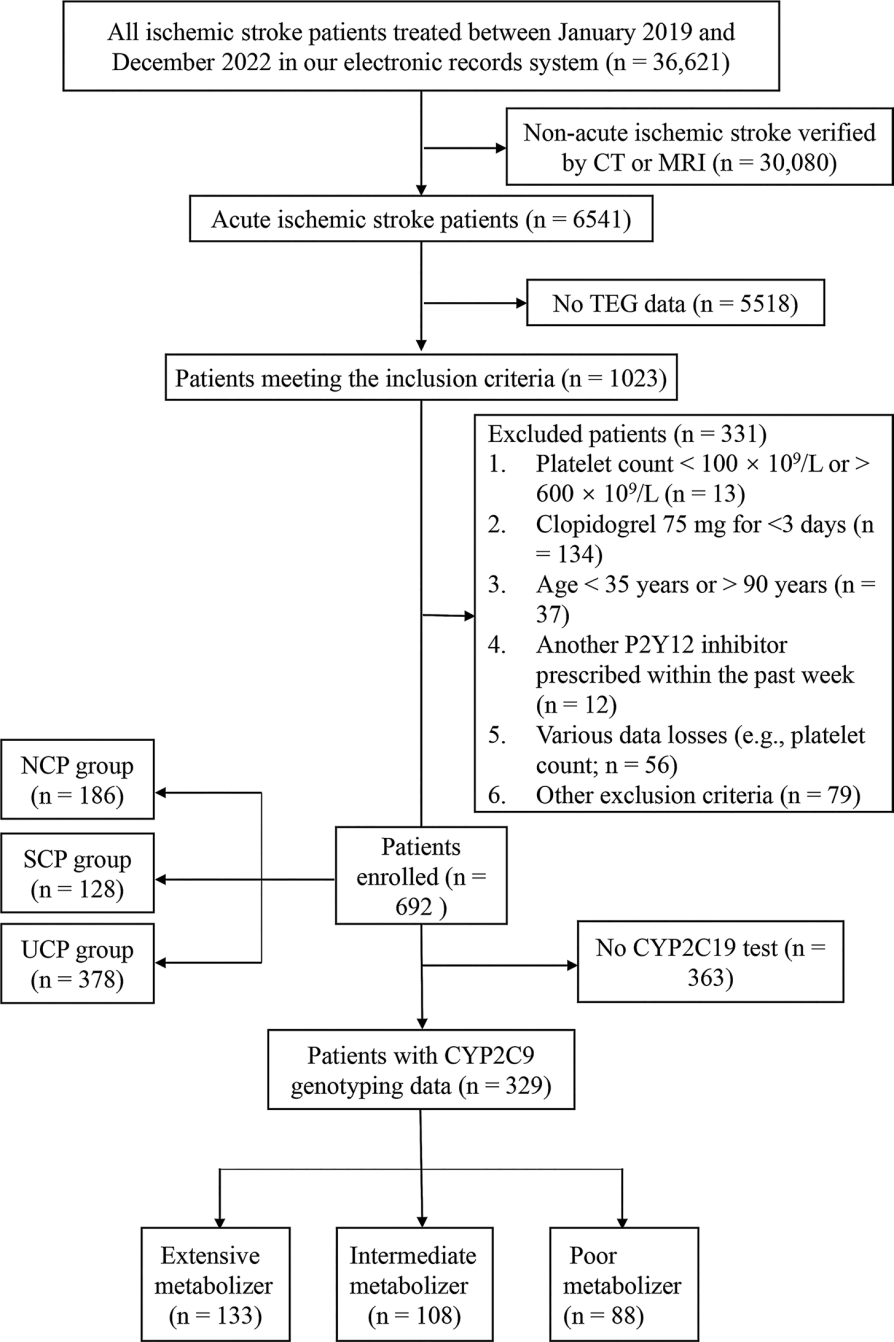
Enrollment illustration of patients. CT, computed tomography; MRI, magnetic resonance imaging; TEG, thromboelastography; NCR, no clopidogrel resistance; CR, clopidogrel resistance

Exclusion criteria included insufficient clinical information, anticoagulant therapy for cardiogenic cerebral embolism or apoplexy caused by brain tumors, absence of clopidogrel use, recent trauma or major surgery, severe dysfunction of the heart, liver, or kidneys, active bleeding, malignancies, respiratory diseases, or immune disorders. The detailed patient selection process is illustrated in Fig. 1.

## Data collection

This study included 692 confirmed AIS patients. Platelet reactivity was assessed using thromboelastography (TEG) after taking clopidogrel at 75 mg/day for 5 ± 2 days. All patients were classified into two groups: CR and non-clopidogrel resistance (NCR). CR was defined as adenosine diphosphate-induced platelet inhibition ratio (ADP-PIR) < 30% after administration of clopidogrel. The demographic, clinical, and laboratory data were collected, including sex, age, personal and family medical history, medication history, complete blood count, lipid profile, glucose levels, biochemical parameters, and glycosylated hemoglobin A1c (HbA1c). Stroke severity was assessed using the National Institutes of Health Stroke Scale (NIHSS) [16, 17].

### Platelet aggregation function assay

ADP-PIR was evaluated using thromboelastography (TEG 5000, Shenzhen Yuepu Corp., China) with the Platelet Mapping (PM) assay. TEG measures the viscoelastic properties of whole blood during clot formation, providing a comprehensive assessment of clotting dynamics, including platelet function, fibrin contribution, and thrombin activity [18]. The PM assay specifically quantifies the degree of platelet inhibition by stimulating platelets with ADP, which targets the P2Y12 receptor inhibited by clopidogrel [19]. A low ADP-PIR (< 30%) indicates high residual platelet reactivity, signifying inadequate inhibition by clopidogrel and thus CR [20, 21]. This threshold of < 30% was selected based on clinical studies, which have correlated it with HTPR and increased risk of recurrent ischemic events [20, 22].

Following a 5 ± 2 day regimen of clopidogrel at 75 mg/day, 3.0 mL of venous blood was drawn into tubes containing heparin and 3.13% sodium citrate. The assay was performed within 2 h of collection to ensure accuracy. The ADP-PIR was tested based on the manufacturer’s instructions of manufacturer and calculated according to the following formula: ADP-PIR (%) = 1 - [(MA_ADP_– MA_fibrin_) / (MA_thrombin_ − MA_fibrin_)] * 100%. In this equation: maximum clot strength (MA) reflects the overall strength of the clot, influenced by platelets, fibrin, and other clotting factors. MA_ADP_ represents the clot strength induced by ADP-activated platelets, indicating residual platelet reactivity in the presence of clopidogrel. MA_fibrin_ measures the contribution of fibrin to clot strength, independent of platelet activity. MA_thrombin_ denotes the maximum clot strength driven by thrombin, reflecting the combined contributions of platelet activation and fibrin polymerization.

### Statistical analysis

Statistical analyses were conducted using SPSS Statistics 27.0 and R software version 4.3.2 (R Foundation for Statistical Computing: https://www.r-project.org). A P-value < 0.05 was considered statistically significant. Continuous variables were expressed as medians (25%, 75%) or means ± standard deviations, and categorical variables were presented as frequencies (percentages).

Initially, the group comparison was performed to screen significant variables (*P* < 0.05) associated with CR. The Chi-Square test was utilized for the analysis of categorical variables. For continuous variables, the independent samples T-test was implemented if the data followed a normal distribution. In cases where the normal distribution assumption was not met, the Mann - Whitney U-test was employed instead. These significant variables were then subjected to least absolute shrinkage and selection operator (LASSO) regression for dimensionality reduction and identification of key predictors. Predictors retained by LASSO regression were subsequently included in a multivariable logistic regression model using bidirectional stepwise selection based on the Akaike Information Criterion (AIC) to construct the final diagnostic model. To evaluate model performance, internal validation was conducted using the bootstrap method (1000 resamples). The model’s discriminatory ability was assessed by calculating the area under the receiver operating characteristic (ROC) curve (AUC). Calibration was evaluated using the Hosmer-Lemeshow goodness-of-fit test, and decision curve analysis (DCA) was employed to determine the clinical utility of the model.

## Result

### Baseline characteristics

The baseline characteristics of the 692 patients are described in Table 1. Among them, 116 patients (16.76%) were categorized into the CR group, while 576 (83.24%) were in the NCR group. Compared with the NCR group, patients in the CR group exhibited higher NIHSS scores (*P* = 0.036) and higher rates of diabetes (*P* = 0.017), proton pump inhibitors (PPIs) (*P* = 0.004), or unstable carotid plaques (*P* < 0.001). Conversely, beta-blockers were significantly lower in the CR group (*P* = 0.045). In terms of laboratory findings, the CR group had significantly lower high-density lipoprotein (HDL) levels (*P* < 0.001) and apoprotein A1 (apo A1) levels (*P* < 0.001), along with higher apoprotein B (apo B) levels (*P* < 0.001). Other variables, such as age and LDL levels, were not significantly different (*P* > 0.05).

**Table 1:** Clinical and laboratory characteristics of patients categorized based on platelet reactivity type

Variable	NCR group (*n* = 576)	CR group (*n* = 116)	*P* value
Clinical characteristics			
Age, years	67.00 (59.00, 75.00)	67.00 (61.00, 78.50)	0.077
NIHSS score, n (%)	3.00 (2.00, 6.00)	4.00 (3.00, 7.00)	0.036
History of CVD, n (%)	128 (22.2%)	30 (25.9%)	0.394
Family history of CVD, n (%)	34 (5.9%)	12 (10.3%)	0.080
Male sex, n (%)	386 (67.0%)	77 (66.4%)	0.895
Hypertension, n (%)	397 (68.9.0%)	77 (66.4%)	0.590
Coronary heart disease, n (%)	74 (12.8%)	13 (11.2%)	0.627
Diabetes, n (%)	187 (32.5%)	51 (44.0%)	0.017
Smoking, n (%)	197 (34.2%)	32 (27.6%)	0.167
Drinking, n (%)	70 (12.2%)	12 (10.3%)	0.583
CCBs, n (%)	201 (34.9%)	48 (41.4%)	0.184
Beta-blockers, n (%)	85 (14.8%)	9 (7.8%)	0.045
ACEIs or ARBs, n (%)	137 (23.8%)	28 (24.1%)	0.935
PPIs, n (%)	188 (32.6%)	54 (46.6%)	0.004
Statins, n (%)			0.784
No statins, n (%)	37 (6.4%)	6 (5.2%)	
Atorvastatin, n (%)	503 (87.3%)	104 (89.7%)	
Rosuvastatin, n (%)	36 (6.3%)	6 (5.2%)	
Laboratory data			
TC, mmol/L	4.33 (3.46, 5.09)	4.15 (3.63, 4.96)	0.488
TG, mmol/L	1.27 (0.94, 1.75)	1.39 (1.11, 1.86)	0.128
HDL, mmol/L	1.02 (0.88, 1.20)	0.94 (0.79, 1.07)	< 0.001
LDL, mmol/L	2.70 (1.95, 3.41)	2.82 (2.04, 3.34)	0.874
FBG, mmol/L	5.10 (4.49, 6.36)	4.99 (4.40, 6.62)	0.493
Serum UA, mmol/L	344.00 (277.00, 412.00)	335.00 (272.00, 393.25)	0.479
HbA1c (%)	5.90 (5.50, 6.90)	6.00 (5.50, 7.20)	0.553
PLT (× 10^9^/L)	239.00 (201.00, 290.75)	238.50 (200.00, 283.75)	0.512
Apo A1, mmol/L	1.23 (1.05, 1.40)	1.07 (0.91, 1.23)	< 0.001
Apo B, mmol/L	0.89 (0.66, 1.12)	1.04 (0.85, 1.41)	< 0.001
Carotid plaques, n (%)			< 0.001
No carotid plaque	181 (31.40%)	5 (4.3%)	
Stable carotid plaques	123 (21.4%)	5 (4.3%)	
Unstable carotid plaques	272 (47.2%)	106 (91.4%)	

* NCR, no clopidogrel resistance; CR, clopidogrel resistance; CVD, cerebrovascular disease; CCBs, calcium channel blocker; ACEIs, angiotensin-converting enzyme inhibitor; ARBs, angiotensin receptor blocker; PPIs, proton pump inhibitor; NIHSS, National Institutes of Health Stroke Scale; TC, total cholesterol; TG, triglyceride; HDL, high-density lipoprotein; LDL, low-density lipoprotein; FBG, fasting blood glucose; UA, uric acid; HbA1C, hemoglobin A1c; PLT, platelet count; Apo A1, apoprotein A1; Apo B, apoprotein B

### Variable selection and key predictors

**Fig. 2:**
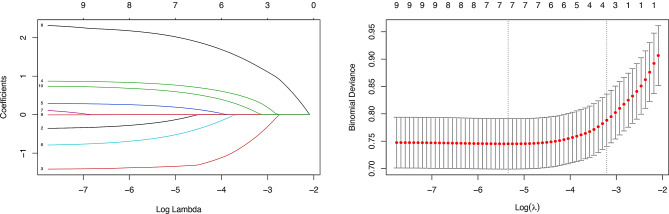
Prediction selection performed by LASSO regression analysis. Figure 2-1 a coefficient of efficiency figure generated against the log (lambda) sequence; Figure 2-2 parameter (lambda) tuning for LASSO regression based on minimum criteria (left dotted line) and 1-SE criteria (right dotted line). In this study, the minimum criteria (left dotted line) were chosen, and 7 variables were screened. LASSO, least absolute shrinkage and selection operator; SE, standard error

In group comparison analysis, 8 variables with *P* < 0.05 were found to be potential predictors of CR. All these variables were further analyzed using LASSO regression with 10-fold cross-validation. The optimal penalty parameter (Lambda.min) was 0.004768949, which selected 7 variables with non-zero coefficients: diabetes, beta-blockers, PPIs, HDL levels, unstable carotid plaque, apo B levels (Figure 2-1, Figure 2-2). Subsequently, stepwise multivariable logistic regression analysis with bidirectional elimination (AIC-based) identified 5 variables as key predictors of C (AIC = 512.78; Table 2): beta-blockers (OR: 0.47, 95% CI: 0.22–1.03, *P* = 0.058), apo A1 (OR: 0.17, 95% CI: 0.07–0.46, *P* < 0.001), apo B (OR: 2.35, 95% CI: 1.23–4.51, *P* = 0.01), unstable carotid plaque (OR: 10.65, 95% CI: 4.18–27.13, *P* < 0.001) and PPIs (OR: 2.09, 95% CI: 1.32–3.31, *P* = 0.002).

**Table 2:** Multivariate logistic regression analyses of the predictor of clopidogrel resistance

Variable	β	OR (95% CI)	*P*
Beta-blockers	− 0.746	0.47 (0.22, 1.03)	0.058
PPIs	0.739	2.09 (1.32, 3.31)	0.002
Apo A1	− 1.749	0.17 (0.07, 0.46)	< 0.001
Apo B	0.855	2.35 (1.23, 4.51)	0.01
Carotid plaques			
Stable carotid plaques	0.214	1.24 (0.35,4.42)	0.742
Unstable carotid plaques	2.365	10.65 (4.18, 27.13)	< 0.001

* PPIs, proton pump inhibitors; TC, total cholesterol; Apo A1, apoprotein A1; Apo B, apoprotein B

### Development of the diagnostic nomogram

**Fig. 3:**
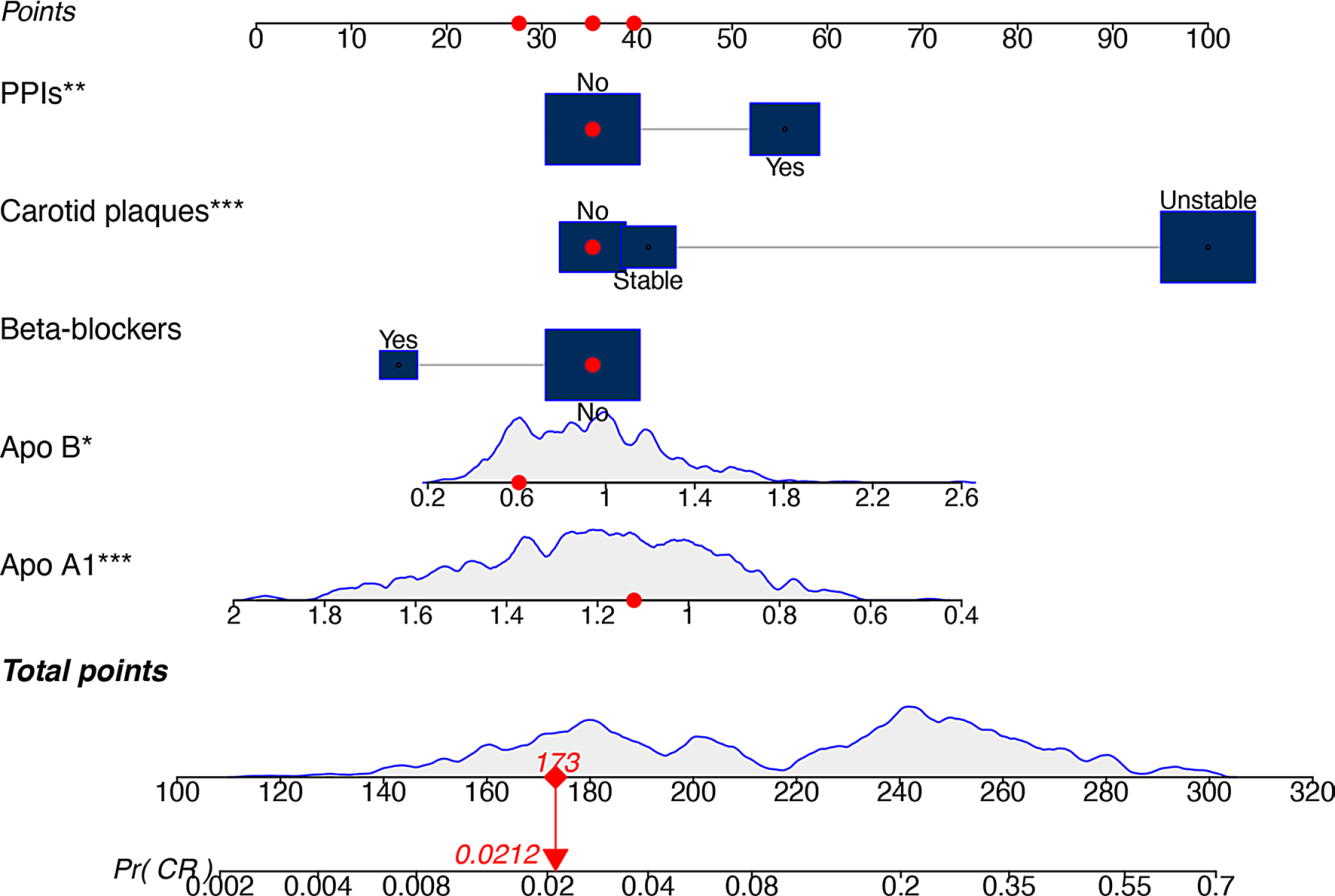
A nomogram for predicting the risk of clopidogrel resistance. PPIs, proton pump inhibitors; Apo A1, apoprotein A1; Apo B, apoprotein B; Pr (CR), Probability of clopidogrel resistance

By using the five identified predictors in multivariable logistic regression, a diagnostic nomogram was developed: unstable carotid plaque, beta-blockers, PPIs, apo A1 levels, and apo B levels (Fig. 3). Unstable carotid plaque had the highest contribution with a maximum score of 100 points, which reflected its strong association with CR. The nomogram may enable clinicians to calculate a total score based on individual predictor values. Then, this score could be mapped onto a probability scale to estimate the likelihood of CR. For instance, a total score of 173 corresponds with a predicted probability of 2.12%.

### Nomogram validation and performance

**Fig. 4:**
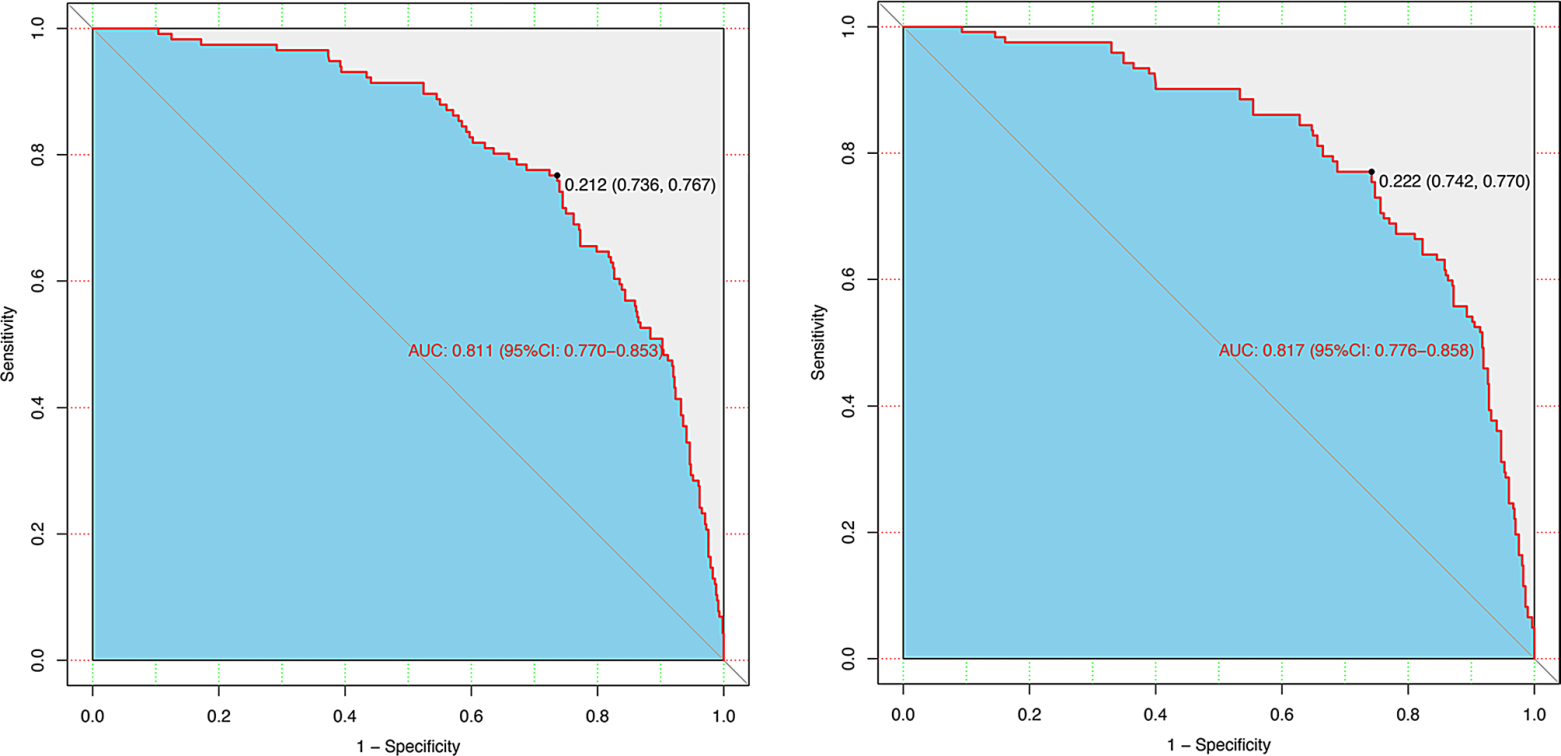
The AUC (representative of the discriminatory power of the model) of the predictive model and the internal validation. AUC of the predictive model (Fig. 4 − 1), and AUC of the internal validation (Fig. 4 − 2). AUC, the area underthe ROC curve

**Fig. 5:**
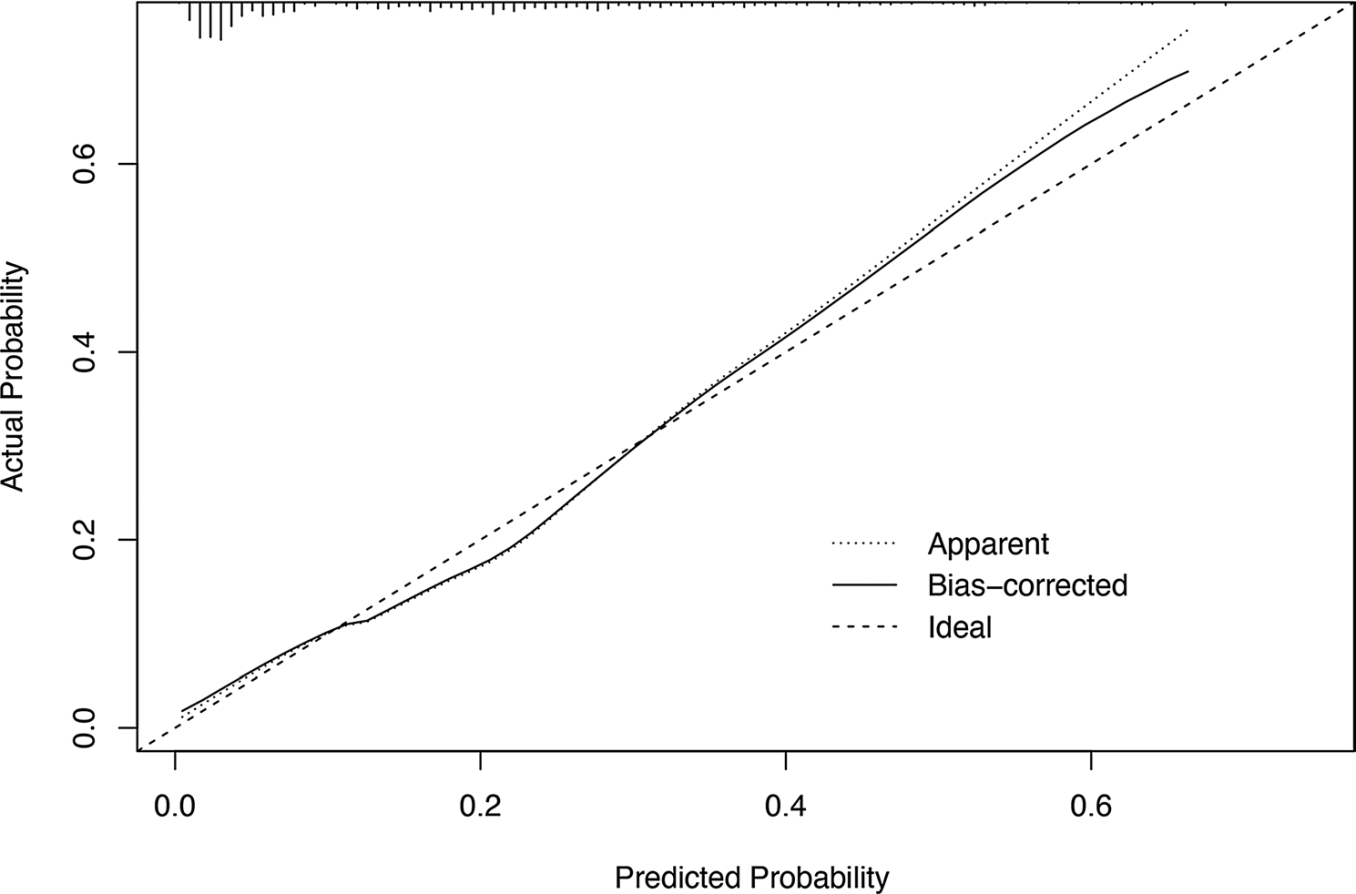
Calibration curves of the nomogram. Based on 1000 repetitions of bootstrapping, the dotted line depicts the overall cohort (*n* = 692), while the solid line reflects the performance of the nomogram after the bias has been corrected by bootstrapping

The performance of this diagnostic nomogram was further validated by the bootstrap method with 1000 repetitions. The primary model showed strong discrimination with an AUC of 0.811 (95% CI: 0.770–0.853) and an optimal cut-off of 0.212 (sensitivity 76.7%, specificity 73.6%) (Fig. 4 − 1). Bootstrap validation confirmed its robustness with an AUC of 0.817 (95% CI: 0.776–0.858) and a cut-off of 0.222 (sensitivity 77.0%, specificity 74.2%) (Fig. 4 − 2). Besides, the calibration analysis showd that the prediction curve closely aligned with the diagonal line, with a H-L goodness of fit test result of *P* = 0.731 and a low Brier score of 0.115, indicating strong consistency between predicted and actual event probabilities and good model fit (Fig. 5).

### Clinical utility of the nomogram

**Fig. 6:**
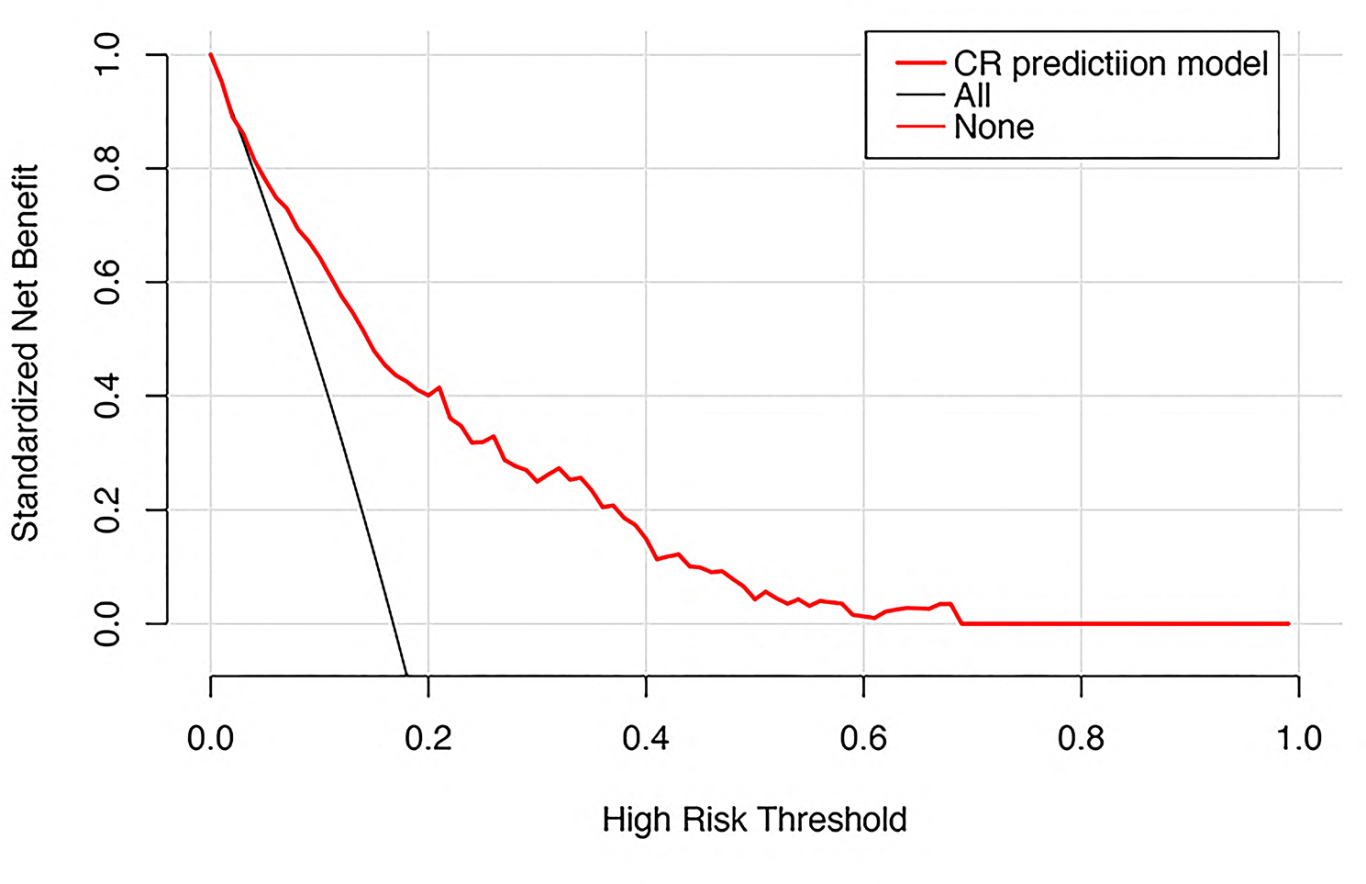
The decision curve of the nomogram. The red solid line stands for the nomogram. (3–68%) On the x-axis are the threshold probabilities, on the y-axis are the net gains

The decision curve analysis in Fig. 6 showed that the net benefit was positive for threshold probabilities between 3% and 68%. Within this range, the model outperformed both the “treat-all” and “treat-none” strategies in identifying a high-risk subset of patients while minimizing unnecessary interventions.

## Discussion

This study identified key predictors of CR in AIS patients, revealing the protective roles of higher apo A1 levels, beta-blockers, risk-enhancing effects of increased apo B levels, PPIs, and unstable carotid plaques. These findings reflected the multifactorial nature of CR influenced by lipid metabolism, drug interactions, and atherosclerosis. By elucidating these associations, the study provided a foundation for targeted strategies to optimize antiplatelet therapy and improve clinical outcomes in AIS patients.

Previous studies have demonstrated that alterations in lipid profiles significantly influence the antiplatelet efficacy of clopidogrel. For example, Shi et al.identified LDL as an independent risk factor for CR [23]. Wadowski et al. reported that low levels of HDL were linked to reduced clopidogrel-mediated platelet inhibition following angioplasty and stenting [24]. Additionally, Pankert et al. observed an association between elevated TC levels and CR, particularly among diabetic patients [25]. In contrast, the present study found that traditional lipid markers, including TC, HDL, and LDL, did not significantly predict CR. This discrepancy may stem from variations in baseline lipid levels or the use of statins among patients. These findings suggested that more specific lipid markers, such as apo A1 and apo B, may provide a more accurate reflection of lipid effects on platelet reactivity and response to antiplatelet therapy.

The current research revealed that apo A1 serves as an independent protective factor against CR, whereas apo B acts as an independent risk factor. Apo A1, the primary component of HDL, enhances clopidogrel’s antiplatelet effects by improving endothelial function through reverse cholesterol transport and reducing platelet activation [26–28]. Conversely, apo B, the main constituent of LDL, promotes platelet aggregation by interacting with platelet surface receptors, thereby reducing clopidogrel efficacy [29]. Furthermore, the apo B/apo A1 ratio, a recognized indicator of cardiovascular risk, was found to be associated with CR [30]. These results have underscored the pivotal roles of apo A1 and apo B in modulating clopidogrel response via lipid metabolism, offering novel insights into the mechanisms driving CR.

Beyond lipid-related factors, drug interactions also play a crucial role in modulating clopidogrel response. This study explored the effects of various medications, including calcium channel blockers (CCBs), beta-blockers, angiotensin-converting enzyme inhibitors (ACEIs) or angiotensin receptor blockers (ARBs), PPIs, statins, and others, on the antiplatelet effect elicited by clopidogrel. The results identified PPIs as an independent risk factor for CR, suggesting they may impair clopidogrel’s efficacy. In contrast, beta-blockers showed a trend toward a protective effect (OR: 0.47, 95% CI: 0.22–1.03, *P* = 0.058), though this did not reach statistical significance. These findings set the stage for understanding how different medications can oppositely influence clopidogrel’s effectiveness.

Building on this, the use of beta-blockers emerged as a potential protective factor against CR in this study. Although the P-value of 0.058 slightly exceeded the conventional significance threshold of 0.05, beta-blockers were incorporated into the diagnostic nomogram for several reasons. A meta-analysis has shown that clinically used beta-blockers could reduce platelet aggregation, particularly nonselective and lipophilic ones [31], possibly by suppressing of beta-adrenergic receptors on platelets [32]. Additionally, prior research reported that patients who received beta-blockers prior to stroke had significantly higher peripheral blood lymphocyte counts [33], while Verdoia et al. found that among acute coronary syndrome (ACS) patients treated with aspirin and ticagrelor after percutaneous coronary intervention (PCI), suboptimal platelet inhibition despite dual antiplatelet therapy (DAPT) was significantly associated with higher neutrophil-to-lymphocyte ratio (NLR) values [34]. Therefore, we hypothesized that beta-blockers may indirectly influence platelet function by reducing sympathetic activity and inflammatory responses (e.g., NLR). Besides, the nomogram was developed using an AIC-optimized multivariable regression model, and the inclusion of beta-blockers reflects their contribution to enhancing the model’s predictive accuracy. Although the statistical evidence from this study remains inconclusive, the biological and clinical context provides a compelling rationale for considering beta-blockers as a protective factor. Further validation of this association requires future studies with larger sample sizes or prospective designs.

PPIs are widely used clinically for the treatment of gastric acid-related diseases, but their combination with the antiplatelet drug clopidogrel in AIS patients has attracted widespread attention. Studies suggested that PPIs may affect clopidogrel metabolism and lower antiplatelet effects, thereby increasing the risk of cardiovascular events [35–37]. Clopidogrel is a prodrug, and the generation of its active form depends on the cytochrome P450 enzyme in the liver, particularly the CYP2C19. Some PPIs may reduce the ability of clopidogrel to convert to its active metabolite [38, 39]. For example, omeprazole and esomeprazole were found to have significant inhibitory effects on the antiplatelet effect of clopidogrel, while pantoprazole affected a relatively small [40, 41]. In a systematic evaluation, the authors reviewed data from several studies and concluded that patients receiving a combination of PPIs had lower inhibitory effects in platelet reactivity compared with clopidogrel alone, suggesting that PPIs may contribute to increased risk of CR [42, 43]. Thus, although PPIs play an important role in the prevention and treatment of gastrointestinal bleeding, clinicians should carefully consider the combination of PPIs in patients using Clopidogrel to avoid potential attenuated antiplatelet effect and increased risk of cardiovascular events [41, 44].

In addition to pharmacological factors, pathological conditions such as carotid plaques also significantly influence the risk of CR. Our result indicated that individuals with unstable carotid plaques had a significantly higher risk for CR. This observation aligned with prior studies by Fusegawa et al., who pointed out that carotid plaques increased platelet aggregability in hypertensive patients. Similarly, Jiaqi Li et al. demonstrated that unstable carotid plaque was significantly related to higher MA on thromboelastography in ischemic stroke patients, indicating stronger platelet activation [45]. Furthermore, some genetic variants were associated with carotid plaque vulnerability, heightened platelet activation, and elevated thromboxane A2 (TXA2) levels at the same time, thus suggesting a mechanistic pathway through whereby unstable plaque promoted thrombotic risk and modulated the variation in antiplatelet response [46]. These findings have emphasized the importance of further investigations into the role of carotid plaque instability in CR and its implications for individualized antiplatelet therapy.

Based on the investigation of crucial factors, we constructed a nomogram to visually represent the intricate regression equation. This nomogram facilitated the visual assessment of the predictive value of the platelet inhibition rate after clopidogrel administration. High performance in the model validation established it as a reliable tool for evaluating the likelihood of CR events after AIS. This would thus, possibly help clinicians determine appropriate antiplatelet therapy, reducing the risk of recurrent strokes and improving outcomes.

Several limitations must be recognized in this study. First, the single-center and retrospective approach of the study undeniably limited the nomogram’s external validity. This could stem from factors such as biases in selecting patients, differences in local prescription habits, and challenges with data reliability, all of which might affect the relationships observed. To tackle this, we intend to strengthen and confirm the nomogram’s external validity by conducting forward-looking studies across multiple centers, utilizing external validation groups, performing sensitivity analyses in varied populations, and benchmarking our findings against existing research. Additionally, the absence of genetic factors—like CYP2C19 polymorphism—might reduce the precision of predictions, suggesting a need for future studies to incorporate a broader range of data dimensions.

## Conclusions

In conclusion, our study indicated that beta-blockers, PPIs, unstable carotid plaque, apo A1, and apo B levels were crucial predictors of CR in AIS patients. Notably, special concern for the influence of lipid levels was attached. Furthermore, the development of a nomogram model would raise clinicians’ diagnostic capability for CR in patients with AIS.

## Data Availability

The data supporting the findings of this study can be accessed from the corresponding author upon a reasonable request.

## Abbreviations

AIS: Acute Ischemic Stroke
CR: Clopidogrel Resistance
NCR: Non-Clopidogrel Resistance
ADP-PIR: Adenosine Diphosphate-Induced Platelet Inhibition Ratio
LASSO: Least Absolute Shrinkage and Selection Operator
OR: Odds Ratio
CI: Confidence Interval
PPIs: Proton Pump Inhibitors
Apo A1: Apolipoprotein A1
Apo B: Apolipoprotein B
HDL: High-Density Lipoprotein
LDL: Low-Density Lipoprotein
NIHSS: National Institutes of Health Stroke Scale
TEG: Thromboelastography
PM: Platelet Mapping
MA: Maximum Clot Strength
MA_ADP_: Clot Strength Induced by ADP-Activated Platelets
MAfibrin: Clot Strength Contributed by Fibrin
MAthrombin: Maximum Clot Strength Driven by Thrombin
ROC: Receiver Operating Characteristic
AUC: Area Under the Curve
DCA: Decision Curve Analysis
H-L: Hosmer-Lemeshow
AIC: Akaike Information Criterion
IS: Ischemic Stroke
HTPR: High On-Treatment Platelet Reactivity
CYP2C19: Cytochrome P450 2C19
ACC/AHA: American College of Cardiology/American Heart Association
TC: Total Cholesterol
CCBs: Calcium Channel Blockers
ACEIs: Angiotensin-Converting Enzyme Inhibitors
ARBs: Angiotensin Receptor Blockers
NLR: Neutrophil-to-Lymphocyte Ratio
ACS: Acute Coronary Syndrome
DAPT: Dual Antiplatelet Therapy
PCI: Percutaneous Coronary Intervention
TXA2: Thromboxane A2
HbA1c: Glycosylated Hemoglobin A1c
